# Adoption of digital payment platforms and trade credit activities among informal firms in Ghana

**DOI:** 10.1016/j.heliyon.2024.e32302

**Published:** 2024-05-31

**Authors:** Mohammed Gbanja Abdulai, Stanley Kojo Dary, Paul Bata Domanban

**Affiliations:** a Department of Economic Studies/School of Economics, University of Cape Coast, Ghana; b Department of Economics, SDD-University of Business and Integrated Development Studies, Ghana; c Department of Development Studies, SDD-University of Business and Integrated Development Studies, Ghana

**Keywords:** Digital payment platforms, Trade credit, Recursive bivariate probit, Informal, Ghana

## Abstract

This study investigates the relationship between the utilisation of digital payment platforms and the decision of informal firms to engage in the demand for or supply of trade credit. Recognizing the pivotal role of trade credit in alleviating financial constraints for informal enterprises, our research employs a recursive bivariate probit model to assess the impact of digital payment platform usage on both the demand for and supply of trade credit among informal firms in Ghana. Leveraging data from the World Bank Enterprise Survey, we find that 13.83 % of informal firms receive trade credit from suppliers, while 26.89 % extend trade credit to customers. Additionally, 49.6 % of firms use digital payment platforms for their business transactions. The study finds that digital payment platforms increase the probability of firms engaging in the demand and supply of trade credit. It argues that digital payments enhance transaction efficiency, convenience, and security, potentially reducing associated transaction costs and facilitating business interactions across distant locations. Various factors, including firm age, maintenance of accounting records, sales volume, owner experience, credit facilities, internet use for social media marketing, and operating hours, significantly influence the decision to engage in trade credit activities. The robustness of our results is confirmed through alternative estimation techniques. Recommendations include policy interventions aimed at promoting the digitalization of informal firms, supported by government investments in digital infrastructure. It is recommended that firms and their suppliers and customers should adopt these digital payment platforms in order to facilitate their use of trade credit in business transactions. A regulatory environment fostering business trust and responsible use of digital payment platforms is crucial, necessitating measures to ensure data protection, security, and ethical conduct within the digital payments’ ecosystem.

## 1 Introduction

Most businesses, whether in developed or emerging nations, have always faced significant challenges in financing their operations. Businesses typically make a variety of investments and transactions that call for either long-term or short-term funding. In order for downstream businesses to get past the obstacle of short-term financing—which arises from the lack of instant cash—they require a type of credit from upstream businesses that helps ease capital limits. In order to free up resources for both short- and long-term needs, trade credit can assist businesses in buying non-perishable items for sale. Accordingly, trade credit arises when there is a delay in payments for products and services received from upstream suppliers [1]. The majority of commercial transactions worldwide use trade credit. These credit agreements are granted by companies or places whose main duty isn't to finance but to sell products and services. Trade credit can be used for a variety of purposes in commercial transactions, as [1] point out. Trade credit can be documented as follows: (i) when buyers of goods or services do not immediately pay suppliers; (ii) when buying firms do not immediately pay suppliers for the supply of goods and services; and (iii) when payment is made in advance of the delivery of goods and services, despite the fact that [1] acknowledge that such arrangements are uncommon. But in recent years, the emergence of e-commerce and mobile money has made the third form of trade credit a widely used system.

Small and medium-sized businesses deal with financial difficulties. A major contributor to the problem of financial constraints faced by firms, especially in developing countries, is the fact that most of the population is financially excluded [2]. According to Ref. [3], the introduction of mobile money has revolutionised the payment environment in developing economies by making financial products and services available to both those with conventional banking accounts and those without them. Approximately 96 % of households in Kenya have at least one M-PESA account, making the country one of the success stories of the mobile money revolution [4]. Prior to the introduction of M-PESA in Kenya, cash (including transfers made through post offices and transportation firms) or the conventional banking system were the two most common ways that individuals and businesses paid for their transactions [5]. Since the introduction of mobile money, Kenya's payment landscape has completely changed, with mobile being the primary payment platform of choice for the majority of families and companies by 2009 [5].

The literature indicates that firms can mitigate the problem of access to finance using trade credit arrangements in a financially constrained environment [6]. Trade credit repayments can be paid with cash or with conventional payment methods, but this option lacks the affordability, convenience, and security of using mobile money [7]. Although giving and receiving credit between businesses is not a novel concept, the introduction of mobile money and its application in commercial dealings is.

In the world of international payments, alternative forms of payment, including digital currencies, mobile wallets, and electronic wallets, continue to be extremely important. Emerging and developing economies are the driving forces behind these trends. In several nations, however, card transactions continue to be the most common method of payment. Not only have certain governments accepted these alternative payment options, but they are also seen as reliable and preferred means of payment. For instance, mobile payment users in Africa are still greater than bank account holders, while alternative payment methods like Alipay are still popular in China and have surpassed PayPal to become the largest global mobile payment platform with more than 300 million registered users [8]. The use of mobile money technologies has grown throughout Africa, beginning with MPESA in Kenya. Evidence has shown that the use of mobile money has brought about increased financial inclusion in developing countries, and Africa in particular [9]. In contrast to conventional payment methods like bank transfers, checks, and cash, Mobile Money has special features such as simplicity of use, minimal transaction costs, accessibility, and ease of payment. Businesses are utilising mobile money for transactions, especially when paying for goods and services and/or receiving payment for them. Despite being the most popular method of payment for consumers and businesses [7], found that the use of cash declines significantly with transaction size. According to a poll, mobile money ranks as the second-most popular payment method, and 80 % of firms use it to pay suppliers and receive payments from clients. Mobile money is chosen over traditional payment methods because of its accessibility, agent customer service, flexibility, and convenience of usage (Beck et al., 2018).

Various studies have examined trade credit dynamics in Africa ([1,[9], [10], [11]]); however, a few have examined how trade credit is determined by innovations in the financial sector ([6,7,12]). The literature on trade credit and digital payments is concentrated in the East African region, where M-PESA originated. In Ghana, digital payments have grown rapidly, resulting especially from the adoption of mobile money services [13]. According to projections, the aggregate value of digital payments in Ghana is expected to reach US$5.87 billion by 2023, with the largest market segment being mobile point-of-sale (POS) payments. Digital payments were predicted to expand at an annual pace of 18.33 % between 2023 and 2027, reaching a market size of US$11.51 billion by that year. Despite the recent trends in mobile money usage and the opportunities it presents to SME's growth, the literature on how it can be used to facilitate innovative finance is rare. This study, therefore, adds to the literature in the following ways: It adds to the growing literature on trade credit in Africa. Also, we provide new evidence on the effect of digital payment platforms on the trade credit activities of informal firms in Ghana. We also assume that informal firms can either demand or supply trade credit, and as such, we analyse both scenarios differently. This study will contribute to policy on finding innovative financing sources for SME's amidst the growing difficulty of accessing credit by firms and, importantly, contribute to the achievement of Sustainable Development Goal 9.

The rest of the paper is structured as follows: Section 2 details the review of both theoretical and empirical literature, while Section 3 discusses the data and sources and the estimation strategies employed. The empirical results and discussion are presented in Section 4, and Section 5 contains the conclusions and recommendations emanating from the research.

## 2 Concept and theories of trade credit

Trade credit comes in a variety of forms. It might also be a prepayment made prior to the delivery of products, which is sometimes referred to in the literature as reverse trade credit. It could be recorded on the books of receiving firms as accounts payable and on the books of supplying enterprises as accounts receivable. As a result, a company may provide or receive a trade credit [1]. The literature identifies three fundamental reasons why businesses utilise trade credit, regardless of their location within the trade credit system. These motivations include operational, commercial, and financial ones.

According to the financial motivation theories, trade credit utilisation is more heavily influenced by the knowledge asymmetry that exists between traditional financing institutions and businesses. According to Ref. [14], providers of products and services are able to execute financial intermediation at a lower transaction cost than traditional financial institutions because they have better information about recipient enterprises than do traditional financing institutions. Because suppliers are closer to businesses and have more frequent interactions with them than traditional financial institutions, they have access to better information. This information allows suppliers to monitor and enforce contracts, establish the creditworthiness of their clients, and decide whether or not credit defaults are intentional. As a result, they can take more appropriate action against defaulters at a lower cost. This makes it possible for non-financial companies with excellent credit worthiness to get credit, assisting other companies that are having trouble obtaining credit [15]. In terms of non-repayment, suppliers also benefit from the products' liquidation and resale. They may also stop supplying future orders to companies that default.

According to the commercial theories, suppliers may use trade credit as a means of implicit guarantees or as a means of price discrimination [16]. According to the notion of price discrimination, companies may engage in indirect pricing discrimination through trade credit. Extending the credit period or raising the discount for on-time payment might be practically translated into a price decrease. In this case, various clients will pay various costs for the identical good [17]. Additionally, because various customers may have varying reservation prices, flaws in the product market may give rise to pricing discrimination [18]. Another way to think of trade credit as a means where suppliers might offer implicit guarantees is. They contend that customers may use the time between delivery and payment to assess the product's quality [19]. Trade credit, according to Ref. [20], is the finest method of product guarantee.

According to operational and transactional theories, economic exchanges include transaction costs that should be taken into consideration when making decisions [21]. Trade credit increases efficiency and lowers costs by separating the exchange of commodities from the payment process. Because the ability to react to changes is supplied elsewhere, this reduces uncertainty about the amount of cash that must be held to satisfy payments [22] and increases operational flexibility [14,23]. According to Ref. [23], trade credit is used to compensate clients who buy products during slow demand times when a company's sales are erratic and cyclical. In this sense, the sellers can lower the storage expenses of the extra inventory that would build up if they maintained steady output by easing the loan terms. Additionally, this saves businesses the money associated with adjusting their output levels.

### 2.1 Empirical review of literature

The literature on the impact of mobile financial services on various aspects of businesses, particularly in developing countries, presents a diverse range of findings and insights. The introduction of mobile money as a payment method has the potential to affect trade credit availability by making repayments easier. According to Ref. [12], using mobile money could facilitate transactions between businesses and clients who are located in different places and lower the cost of transactions related to financial transactions. According to Ref. [24], homes having access to mobile money are able to conduct transactions up to 100 km away from their location. Their findings suggest that mobile money may help shorten trade credit payback delays. This suggests that, regardless of the supplier's distance from the business, suppliers are more inclined to grant trade credit to companies that have access to mobile money on the grounds that they can shorten the repayment time. Furthermore, by lowering transaction costs, mobile money can help businesses become more liquid and allow business owners to offer their clients credit for goods and services.

[7,25] provide insightful analyses of the complex link that exists between the use of mobile money and the availability of trade credit for enterprises [25]. demonstrate the positive relationship between increased access to trade credit and the use of mobile money for supplier payments in their dynamic general equilibrium model. This implies that the availability of trade credit is facilitated for enterprises that use mobile money. To elaborate [7], explore the underlying mechanisms in a more comprehensive way. Their research demonstrates a strong positive correlation between the use of mobile money and the availability of trade credit for businesses, highlighting the risk-reduction feature of mobile money as a factor that favourably affects credit market valuation.

[26] extend our understanding of the impact of mobile money services, specifically on microenterprises in Kenya. Their comprehensive investigation shows that a number of mobile money services, such as mpesa for business, pochi la biashara, digital payment services, savings and loan services, and others, together account for a sizable percentage of the difference in trade credit access between enterprises in the Kamukunji sector. The complex link that their research uncovered highlights the diverse ways in which Safaricom Ltd.'s mobile money services affect microenterprises' ability to obtain trade credit.

In the context of mobile money usage [[27], [28], [29]], offer crucial viewpoints on the performance of businesses, especially small and medium-sized firms (SMEs) [27]. sheds light on the real-world usage of mobile money in business-to-business (B2B) and customer-to-business (C2B) transactions by concentrating on the positive effects of mobile money transfers on the sales volumes of SMEs. The results of [28] highlight how the use of mobile money improves the growth trajectory of small and medium-sized enterprises (SMEs), demonstrating the critical role that this financial technology plays in promoting company expansion. Furthermore [29], offers a comprehensive understanding of the dynamic relationship between mobile money and SME performance through its nuanced categorization of mobile money usage based on accessibility, affordability, flexibility, and convenience. This highlights the various ways in which these factors positively impact the profitability of firms.

[30] examines how Kenyan households in the unofficial sector use savings and lending services, identifying important drivers and patterns. The survey emphasises the variety of reasons people use financial services to save money, from starting new enterprises to covering everyday expenses. This viewpoint expands our knowledge of mobile money's integration with financial services in the informal sector by highlighting the role it plays in offering financial solutions customised to the particular requirements of individuals in this sector [31]. investigate the impact of mobile financial services on the unorganised sector using data from emerging nations. They discover that the scale of the informal sector is negatively impacted by mobile banking services. They contend that the effect could have several causes, including the potential for inspired development of businesses already operating in the formal sector and an increase in the profit level of informal firms, which would lessen the subsistence restrictions typical of entrepreneurship in the informal sector [32]. introduces the dimension of external finance into the discussion of mobile money usage by firms. The study finds a significant relationship between the use of mobile money and businesses' ability to obtain external financing. This new finding is consistent with a larger body of research on trade credit and implies that entrepreneurs that have easier access to capital are more likely to offer trade credit to clients. The study adds to our understanding of the complex ways in which mobile money might act as a spur for companies looking for outside funding.

The works of [6,33] add to our understanding of the relationship between corporate investment and mobile money usage. According to Ref. [33], there is a positive link between the use of mobile money and businesses' investments in fixed assets. This phenomenon is explained by the lower transaction costs and enhanced creditworthiness that mobile money enables. According to Ref. [6], there is a significant relationship between the use of mobile money and receiving items from suppliers on credit as well as giving customers credit. Our comprehension of how mobile money might facilitate capital investment and trade ties among enterprises is further enhanced by these findings [34]. using data on firms from rural locations indicated that the use of mobile money by firms broaden their customer base given that majority of people have access to a mobile money account. They concluded that the ease of access, convenience and lower transaction cost associated with mobile money use relative to other platforms has led to a growth in firms located in rural areas.

[35,36] contribute perspectives on the resilience of firms in the face of challenges, highlighting mobile money as a potential mitigating factor. According to Ref. [35], mobile money can facilitate financial transactions that help smaller businesses deal with the COVID-19 pandemic's impacts. Mobile money becomes an affordable, convenient, and broader consumer base, making it a resilient tool for small enterprises. This theory is supported by Ref. [36], which used a sample of 285 Cameroonian businesses and found that mobile money receipts and payments were responsible for a sizable amount of the overall variations in business profitability. All of these studies point to how important it is for businesses to implement mobile money in order to improve their financial performance and resilience.

Other firm specific factors affecting the demand and supply of trade credit are documented in the literature [37]. finds that both internal and external factors influenced a firm's demand for trade credit. They highlight internal factors including, inventory turnover, availability of collateral, payment period and reorder point. While some of the external factors include, customer monopoly, bank availability and living in metropolitan areas. Businesses with higher profitability and liquidity ratios make less demands for trade credit, according to Ref. [38]. They demonstrated how trade credit demand is negatively impacted by return on asset, fast, and current ratio. In a different study [39], demonstrated that inventory turnover has a favourable impact on demand for trade credit and that the number of trade credit increases when accounts payable payment periods grow. The results of the [40] study indicated that businesses with limited liquidity would request less trade credit [41]. found that Spanish businesses with limited credit have a greater reliance on trade credit. In a similar vein [42], claimed that during a crisis, accounts payable rise for limited businesses [43]. discovered in another study that companies facing financial difficulties are more inclined to incorporate trade credit into their overall growth strategy. According to Ref. [44], trade credit (also known as accounts payable) is considerably and negatively impacted by both long-term debt and profitability, whereas trade credit is positively impacted by short-term debt. Interestingly, trade credit replaces long-term debt even if it appears to supplement other short-term debt.

There is a growing body of evidence on the role played by digital payment systems in the demand for and the supply of trade credit. The literature in developing countries have mainly focused on mobile payments in Kenya most especially when it is the origin of mobile payments in Africa with the introduction of the M-PESA. Studies on trade credit in Ghana seem limited and more especially trade credit activities among informal firms. This study thus contributed to the literature on trade credit among informal firms by testing the following hypothesis.

H1 Digital payment platforms increase the probability of trade credit demand among informal firms in Ghana.

H1 Digital payment platforms increase the probability of trade credit supply among informal firms in Ghana.

## 3 Methods and materials

### 3.1 Data

The study utilised cross-sectional data on informal firms collected by the World Bank for the 2022 round. Because informal firms are by definition excluded from many official surveys and statistics, the World Bank's Informal Sector Enterprise Surveys are initiatives to collect data based on a sampling approach and a questionnaire designed to meet the challenges of monitoring the activity of informal enterprises. The survey uses a geographic sampling technique called the Adaptative Cluster Sampling (ACS) that ensure that a representative sample of firms in the informal sector are selected. ACS posits that informal business activity tends to be concentrated in specific regions and leverages the insights gained from identifying certain informal businesses to adjust the likelihood of selecting primary sampling units (PSUs) in order to better capture clusters of additional informal businesses. By accounting for this adjusted probability of PSU selection, ACS can estimate the total population of informal businesses, thus enabling any follow-up survey of these businesses to be deemed representative [45]. Adaptative cluster sampling involves unequal probability of sampling. However, the standard Horvitz-Thompson and Hansen-Hurwitz estimators are modified to provide unbiased estimates of finite population parameters along with unbiased variance estimators. It's especially useful when the informal businesses are missing from standard sampling frames and are geographically clustered. The ACS is also more efficient than other sampling methodologies when the population is large and geographically dispersed as in the case of informal firms in Ghana [45] Since 2016, the polls have been conducted using a standardised instrument that has been applied consistently in a number of economies. All unregistered firms in the specified locations are covered by the surveys, which are intended to be geographically representative of particular metropolitan areas [45]. The current round for Ghana has a total of 4571 informal firms included in the survey. Further information about the data and their methodology can be obtained at [45]. Further analysis of the data is based on the complete case analysis as proposed by Ref. [46]. This is due to the fact no systemic pattern in the missing variables that would mean no randomness in the data missing and will therefore not bias the results of the study. Responses that were coded as “I don't know (−9) and refusal to answer (−8) were treated as missing”

Table 1 shows the definitions and descriptions of the variables used in the study. We use two measures of trade credit (trade credit demand and trade credit supply). A firm is said to demand trade credit if it receives trade credit for the purchase of inputs from upstream suppliers. Firms are also said to be involved in trade credit supply if they extend trade credit to downstream customers for the sale of goods. The data indicates that about 13.83 percent of informal firms receive trade credit from suppliers, and another 26.89 percent supply trade credit to customers.

**Table 1:** Description of variables.

Variable	Description	Obs	Mean (%)
TC demand	Fims are asked if they engage in buying goods from supliers ahead of time and paying later. It is coded as 1 if firm purchases inputs on credit, 0 otherwise	4473	13.83
TC Supply	Fims are asked if they engage in selling goods to customers and receiving payments later. This is coded as 1 if firm sells goods on credit, 0 otherwise	4514	26.89
Digital money	This is measured using a binary variable. This is coded as 1 if firm uses mobile money for purchase and sale of goods and services 0 otherwise [6]	4520	49.60
Age	Number of years firms has been in operation [6]	3999	7.66 years
Account	1 if firm keeps account of company records and zero otherwise [6]	4419	28.22
Sales	This is measured as the volume of sales of the firm in the previous year.	2665	47946.78
Experience	Number of years owner has operated business	3957	7.94 years
Loan	1 if firm has a credit facility, 0 otherwise.	4571	10.92
Internet	1 if firm uses internet and social media for marketing, 0 otherwise.	4564	9.16
Operation hours	Number of hours firm usually operate in a week	4564	60.53 h
*Business type*			
Retail	Indicates the sector of production the firms operate in. 0 indicates retail (the base category), 1 indicates manufacturing and 2 indicates Services	2939	64.30
Manufacturing		658	14.40
Services		974	21.31
*Education*			
No education	Indicates the educational level of the owner of the business. 0 indicates no education, 1 indicates basic education, 2 indicates secondary and 3 indicates tertiary education	664	15.25
Basic		1921	44.12
Secondary		1371	31.49
Tertiary		398	9.14

We measure digital money as a binary to indicate whether or not a firm uses digital payment platforms for business transactions. The survey asks respondents if they used mobile money for business transactions. The data indicates that 49.6 percent of informal firms make use of digital payment platforms in business transactions. Other variables controlled for the models include the age of the firm, the keeping of accounting records, the volume of sales, the experience of the owner of the firm, loan facility, the use of the internet and social media for marketing purposes, and the number of hours worked in a month. All variables are defined in Table 1. On records keeping, the table indicates that about 28.22 percent of informal firms in Ghana keep accounting records. This could be because most informal businesses are usually retail-based businesses that, by practice, mostly restock when they run out of stock and usually do not keep accounting records. From Table 1 and it is shown that about 64.3 percent of firms used in the study were in the retail business, while another 14.4 percent were in manufacturing, and those in the services sector were 21.31 percent.

### 3.2 Econometric model

The theoretical framework for this study indicates that the decision of informal firms to engage in the demand and supply of trade credit could be influenced by the availability of digital payment platforms. Conversely, the decision to demand or supply trade credit could also influence a firm's decision to subscribe to digital payment platforms. Such a framework has two dependent variables, which are binary, and as such, the use of digital payment platforms becomes endogenous to the estimation of the demand and supply of trade credit. Hence, it is expected that latent variables could influence the propensity to embrace digital payment platforms as well as the inclination to engage in trade credit [12]. Furthermore, a possible simultaneity exists in the association between the digital payment platform adoption and the trade credit ([7,47]). Following [48], we use a recursive bivariate probit model and decomposition approaches in the estimation. A recursive bivariate allows the estimation of two equation in two steps, where in the first step we determine what factors influence adoption of digital payment platforms and in the second step, we estimate the decision to engage in trade credit using the predicted value of the digital payment platforms as an independent variable. The recursive bivariate probit model assumes a linear specification between predictors and latent variables, errors follow bivariate normal distribution and instrument exogeneity and relevance. This model corrects for endogeneity by simultaneously estimating the decision to use digital payment platforms and trade credit. It estimates the two probit models with bivariate normally distributed error terms. The correlation between the error terms captures the endogeneity. The limitations of the model lie in the fact that if an exclusion variable is not accounted for, it becomes weakly identified and also the treatment variable must be binary. We begin with a specification of two structural equations (1), (2) as:

(1) $$y_{1}^{*}=x_{1}^{\prime }\beta_{1}+\delta y_{2}+\mu_{1},y_{1}=m\;if\;k_{m-1}\leq y_{1}^{*}<k_{m}\;for\;m=1,2\ldots ‥n$$

(2) $$y_{2}^{*}=x_{2}^{\prime }\beta_{2}+\mu_{2},y_{2}=1\;if\;y_{2}^{*}>0$$

(3) $$\text{Given}\;\text{that}\;\left(\begin{array}{c} \mu_{1}\\ \mu_{2} \end{array}\right)∼N\left[\left(\begin{array}{c} 0\\ 0 \end{array}\right),\left(\begin{array}{cc} 1 & \rho \\ \rho & 1 \end{array}\right)\right]$$

Where $\mu_{1}$ and $\mu_{2}$ in equation (3) are jointly normally distributed and therefore may be correlated. The latent endogenous variables of the model are $y_{1}^{*}$ and $y_{2}^{*}$ observable at their binary realisations. In this situation, $y_{1}$ is binary and represents the demand for trade credit or the supply of trade credit and $y_{2}$ is also binary and represents the decision to use a digital payment platform. If there is enough variance in the explanatory variables, the recursive probit model—as opposed to the linear recursive model—can be identified without the need for an exclusion restriction. To ensure the identification of the model, an exclusion restriction is performed using a variable, payment of some employees electronically. The justification for the use of the exclusion variable in the identification model, is due to the fact that firms who will want to pay employees might need to use mobile money for those purposes. However, the exclusion variable does not have a direct effect on the demand or supply for trade credit. Other independent variables included in the model as control variables are explained in Table 1.

Since no clear economic interpretation can be ascribed to probit models, the significance of variables is usually assessed through the calculation of marginal effects. The Average Treatment Effect (ATE) of the binary endogenous regressor ($y_{2}$) is the average treatment effect of digital payment platform use on the demand or supply of trade credit. According to Ref. [49], the ATE can be expressed as in equation (4):

(4) $$ATE=\frac{1}{n}\sum_{i=1}^{n}{\psi (x}_{1}^{\prime }\beta_{1}+\delta )-\psi \left(x_{1}^{\prime }\beta_{1}\right)$$

The Average Treatment Effect on the Treated (ATET) can also be expressed as shown in equation (5):

(5) $$ATET=\frac{1}{n_{2}}\sum_{i=1}^{n_{2}}\psi \left(\frac{x_{1}^{\prime }\beta_{1}+\delta -{\delta \rho x}_{2}^{\prime }\beta_{2}}{\sqrt{1-\rho^{2}}}\right)-\psi \left(\frac{x_{1}^{\prime }\beta_{1}-{\rho x}_{2}^{\prime }\beta_{2}}{\sqrt{1-\rho^{2}}}\right)\forall y_{2i}=1$$

While the Average Treatment Effect on Conditional Probability (ATEC) is expressed as in equation (6):

(6) $$ATEC=\frac{1}{n_{2}}\sum_{i=1}^{n_{2}}\psi \left(\frac{\psi_{2}\left(x_{1}^{\prime }\beta_{1}+\delta {\rho x}_{2}^{\prime }\beta_{2}\right)}{\psi \left(x_{2}^{\prime }\beta_{2}\right)}\right)-\left(\frac{\psi_{2}\left(x_{1}^{\prime }\beta_{1}-x_{2}^{\prime }\beta_{2}-\rho \right)}{\psi \left(-x_{2}^{\prime }\beta_{2}\right)}\right)$$

We can decompose the marginal effects into direct and indirect effects as suggested by Ref. [49]. The marginal effects for continuous variables can then be computed as shown in equation (7) following [49].

(7) $$ME=\frac{\gamma \;\mathrm{Pr}}{\gamma \left(\begin{array}{c} x_{1T}\\ x_{1E} \end{array}\right)}=\frac{\gamma \;\mathrm{Pr}}{\frac{\gamma x_{1T}}{direct\;effect}}+\frac{\gamma \;\mathrm{Pr}}{\frac{\gamma x_{1E}}{indirect\;effect}}$$

The marginal effects for discrete variables can also be computed as indicated in equation (8).

(8) $$ME=\frac{{|\mathrm{Pr}|}_{x_{1T}=1}-{|\mathrm{Pr}|}_{x_{1T}=0}}{direct\;effect}+\frac{{|\mathrm{Pr}|}_{x_{1E}=1}-{|\mathrm{Pr}|}_{x_{1E}=0}}{indirect\;effect}$$

The empirical model could then be stated as follows in equations (9), (10).

(9) $$\mathrm{Pr}\left({TC}_{i}=1\right)=\beta_{0}+\beta_{1}\hat{{dm}_{i}}+\beta_{2}{age}_{i}+\beta_{3}{account}_{i}+\beta_{4}{busitype}_{i}+\beta_{5}{sales}_{i}+\beta_{6}{experience}_{i}+\beta_{7}{education}_{i}+\beta_{8}{loan}_{i}+\beta_{9}{internet}_{i}+\beta_{10}{operationhours}_{i}+\mu_{1}$$

(10) $$\mathrm{Pr}\left({dm}_{i}=1\right)=\beta_{0}+\beta_{1}{age}_{i}+\beta_{2}{account}_{i}+\beta_{3}{busitype}_{i}+\beta_{4}{sales}_{i}+\beta_{5}{experience}_{i}+\beta_{6}{education}_{i}+\beta_{7}{loan}_{i}+\beta_{8}{internet}_{i}+\beta_{9}{operationhours}_{i}+\beta_{10}{Pay}_{i}+\mu_{2}$$

## 4 Results and discussions

This section presents the results of the effect of digital money on the demand for and supply of trade credit activities among informal firms in Ghana. A recursive bivariate probit model is used to estimate the relationship. Additionally, a classic probit model is used as a test for robustness. Table 2 shows the results of the pairwise correlation matrix between the variables used in the study. The correlation matrix indicates that digital money has a significant positive correlation with both trade credit demand (0.124) and trade credit supply (0.253), respectively. The results of the correlation analysis further indicate that the issue of multicollinearity should not be a major concern.

**Table 2:** Pairwise correlations.

Variables	(1)	(2)	(3)	(4)	(5)	(6)	(7)	(8)	(9)	(10)	(11)
(1) tcinward	1										
(2) tcoutward	0.376*	1									
	(0.000)										
(3) dm	0.124*	0.253*	1								
	(0.000)	(0.000)									
(4) age	0.089*	0.149*	0.114*	1							
	(0.000)	(0.000)	(0.000)								
(5) account	0.169*	0.189*	0.374*	0.152*	1						
	(0.000)	(0.000)	(0.000)	(0.000)							
(6) sales	−0.007	0.028	0.019	0.009	0.028	1					
	(0.736)	(0.145)	(0.318)	(0.638)	(0.154)						
(7) experience	0.109*	0.130*	0.092*	0.817*	0.177*	0.005	1				
	(0.000)	(0.000)	(0.000)	(0.000)	(0.000)	(0.803)					
(8) loan	0.262*	0.197*	0.197*	0.102*	0.284*	−0.006	0.155*	1			
	(0.000)	(0.000)	(0.000)	(0.000)	(0.000)	(0.738)	(0.000)				
(9) internet	0.147*	0.145*	0.222*	0.009	0.226*	−0.007	0.021	0.139*	1		
	(0.000)	(0.000)	(0.000)	(0.557)	(0.000)	(0.720)	(0.187)	(0.000)			
(10) hours	0.047*	0.098*	0.077*	0.057*	0.039*	0.005	0.128*	0.163*	0.048*	1	
	(0.003)	(0.000)	(0.000)	(0.001)	(0.014)	(0.802)	(0.000)	(0.000)	(0.002)		
(11) education	−0.06*	−0.01	0.220*	−0.10*	0.225*	0.018	−0.14*	0.083*	0.214*	0.012	1
	(0.000)	(0.691)	(0.000)	(0.000)	(0.000)	(0.344)	(0.000)	(0.000)	(0.000)	(0.461)	
(12) bustype	−0.08*	−0.06*	0.102*	0.073*	0.046*	0.033	0.134*	0.006	0.134*	0.068*	0.101*
	(0.000)	(0.000)	(0.000)	(0.000)	(0.002)	(0.089)	(0.000)	(0.680)	(0.000)	(0.000)	(0.000)

Standard errors in parentheses.

*p < 0.05, **p < 0.01, ***p < 0.001.

### 4.1 Effect of digital payment platforms on the demand for and supply of trade credit among informal firms

Using a full-information maximum likelihood function, we estimate a recursive bivariate probit of the effect of digital payment platforms on the demand and supply of trade credit among informal firms in Ghana. Table 3 presents the results of the recursive bivariate probit. The significant Wald Chi squares in both models indicate that the estimated models produced good fits for the data. It also tests against the null hypothesis of zero covariance between the error terms of the recursively estimated models. The result of the rho indicates that the errors of the recursively estimated models for demand and supply of credit and digital payment platforms are correlated, which thus allows for an estimation of the recursive bivariate probit model.

**Table 3:** Results of recursive bivariate probit.

Variables	Trade credit demand		Trade credit supply	
	Trade credit	Digital money	Trade credit	Digital money
dm	1.054*** (0.372)		0.486*** (0.033)	
Age	−0.005 (0.008)	0.006 (0.008)	0.039*** (0.011)	0.005 (0.008)
Account	0.092 (0.157)	0.696*** (0.075)	0.209* (0.115)	0.699*** (0.074)
Business type				
Manufacturing	−0. 119 (0.096)	0.291*** (0.089)	0.105 (0.081)	0.279*** (0.089)
Services	−0.510*** (0.094)	0.260*** (0.081)	−0.445*** (0.087)	0.238*** (0.080)
Sales	−0.001 (0.001)	0.001*** (0.001)	0.001*** (0.001)	0.001*** (0.001)
Experience	0.007*** (0.001)	−0.007 (0.008)	−0.013 (0.009)	−0.005 (0.008)
Education				
Basic	−0.317*** (0.105)	0.157* (0.093)	−0.076 (0.101)	0.171* (0.094)
Secondary	−0.724*** (0.145)	0.762*** (0.109)	−0.049 (0.145)	0.7811*** (0.108)
Tertiary	−0.471** (0.185)	0.639*** (0.157)	−0.023 (0.176)	0.662*** (0.157)
Loan	0.926*** (0.143)	0.295** (0.135)	0.669*** (0.114)	0.343* (0.130)
Internet	0.285** (0.137)	0.542*** (0.136)	0.298*** (0.114)	0.576*** (0.137)
Operation hours	−0.001 (0.001)	0.003* (0.001)	0.004*** (0.001)	0.003** (0.001)
Pay		0.413*** (0.111)		0.475*** (0.106)
C	−1.314*** (0.124)	−0.949*** (0.106)	−1.283*** (0.140)	−0.979*** (0.126)
/atanrho	−0.480* (0.286)		−0.075 (0.198)	
Wald test of rho = 0:	2.821***		1.143***	
Observations	2038		2038	

Robust Standard errors in parentheses.

*p < 0.05, **p < 0.01, ***p < 0.001.

#### 4.1.1 Trade credit demand

The result indicates that demand for trade credit is influenced by the use of digital payment platforms, businesses that are into manufacturing, the experience of the owner or manager of the firm, the education of the owner, and access to loan facilities. The use of digital payment platforms is positively correlated with the demand for trade credit among informal firms. Firms that used digital payment platforms were more likely to receive input from suppliers on credit and pay at a later date than firms that did not use digital payment platforms. Businesses that employ digital payment platforms probably see improvements in transactional efficiency and convenience. The payment procedure is streamlined by digital payment platforms, which makes it quicker and easier [12]. Due to their dependable and prompt payment methods, suppliers may be more inclined to grant trade credit to these companies as a result of their efficiency. Digital payment systems frequently have transparency and security built in. This has the potential to increase buyer-supplier trust and increase the willingness of suppliers to offer products or services on credit to businesses that use digital payment platforms. Additionally, the digital record of transactions gives the businesses more legitimacy, which increases their appeal to suppliers for trade credit agreements. This result is consistent with the findings of [6,7,26], who all find that trade credit is positively associated with mobile money use in Kenya. They all argue that the use of mobile money for business transaction allowed firms to gain access to external financing including trade credit arrangements.

The results further indicate that manufacturing firms were less likely to demand inputs on credit from suppliers than firms involved in retail businesses. For their manufacturing operations to continue, manufacturing companies frequently require a consistent supply of inputs and raw materials. Because of their possibly more consistent production schedules and inventory control procedures, these businesses are able to plan and buy inputs more methodically. They might therefore require less instant financing from vendors. Having more experience in the management of a business is associated with a higher likelihood of purchasing inputs from suppliers on terms of trade credit. Experienced managers or business owners are more likely to have developed long-lasting relationships with suppliers. These connections could be based on reliability, trust, and a track record of successful transactions. Experienced business managers who have a good history with suppliers may be eligible for better credit terms. A more favourable creditworthiness profile may also be linked to business management experience. Suppliers and lenders frequently evaluate a company's creditworthiness prior to extending trade credit. Suppliers are more likely to grant trade credit to managers who have a track record of effective business operations and financial accountability since they are seen as lesser credit risks. This contradicts the work of [6].

Also, businesses that already have credit facilities with financial institutions are more likely to purchase goods from upstream suppliers on trade credit terms than those with credit facilities. Possessing credit facilities could improve a company's ability to bargain with suppliers upstream. Businesses having established credit agreements with financial institutions may receive more favourable trade credit terms from suppliers, as this shows a degree of trust and confidence in the company's financial health.

Table 3 further shows that the use of digital payment platforms is influenced by the keeping of accounting records, the manufacturing and service firm, firm sales, the education of the owner, the use of the internet and social media for marketing purposes, and the payment of some employees electronically. The results indicate that the decision to use digital payment platforms is positively influenced by all the variables mentioned above.

#### 4.1.2 Trade credit supply

Trade credit supply occurs when firms sell goods to downstream consumers for payment at a later date. Table 3 shows the supply of trade credit is influenced by the use of digital payment platforms, the age of the firm, services firms, sales, education, credit facilities, the use of the internet and social media for marketing, and the number of hours worked per month. The use of digital payment platforms is positively correlated with the supply of trade credit among informal firms. Firms that used digital payment platforms were more likely to sell goods to downstream consumers and receive payments at a later date than firms that did not use digital payment platforms. The payment process is frequently streamlined by digital payment platforms, which makes it quicker and more effective. Because digital payment systems enable informal businesses to manage their accounts receivable more effectively, this efficiency may encourage these businesses to provide trade credit to downstream consumers. It is simpler for businesses to issue credit when transactions are dependable and swift. Additionally, by giving customers more flexible payment options, informal businesses that use digital payment platforms may be able to acquire a competitive edge. This benefit has the potential to increase clientele and cultivate a sense of loyalty, which will boost revenue. Businesses may be more likely to offer trade credit in order to satisfy customers and maintain their competitiveness. The result is in line with the findings of [6,50].

Older firms were more likely to supply trade credit than relatively younger firms. Customers of older businesses frequently develop enduring relationships with them. Trade credit agreements can be negotiated and implemented more easily by older businesses when these partnerships are based on mutual trust and understanding. An aura of dependability is fostered by the familiarity that older businesses have with their clientele. Firms in the services subsector were less likely to be involved in the supply of trade credit relative to firms in the retail sector. Businesses that focus on providing services frequently offer non-tangible and intangible products and services, including software solutions, professional services, and consulting. When compared to tangible products offered in the retail sector, these services might not lend themselves to trade credit as easily. Service companies may find it more difficult to set up and carry out trade credit agreements if they don't sell physical goods.

Sales volumes of a firm are associated with a higher likelihood of supply of trade credit among informal firms in Ghana. Businesses with greater sales volumes frequently possess greater capacity for cash flow. Because of their strong financial position, they may offer trade credit to clients without endangering their capacity to pay other debts. Strong cash flow gives you the liquidity you need to give buyers credit conditions. The results further indicate that firms with credit facilities were more likely to supply trade credit relative to firms with no credit facility with financial institutions. Moreover, firms that use the internet and social media for marketing purposes were more likely to be involved in the supply of trade credit. Also, firms that worked longer hours were more likely to be involved in the supply of trade credit relative to firms that worked fewer hours.

Table 3 shows that the decision to use digital payment platforms is positively influenced by the payment of employees electronically, keeping of accounting records, manufacturing a service firm, sales volume, educational status of the owner, credit facility, and the use of the internet and social media for marketing purposes.

#### 4.1.3 Treatment effects of the use of digital payment platforms on the demand and supply of trade credit

Table 4 shows the average treatment effect of the use of digital payment platforms on the demand and supply of trade credit among informal firms in Ghana. For all three of the calculated treatment parameters, the estimated parameters are statistically significant. The average treatment effect of the use of digital payment platforms on the demand for trade credit is about 0.240, indicating that the use of digital payment platforms increases the probability of demand for trade credit by about 24 percent relative to firms that do not use digital payment platforms. Also, the use of digital payment platforms increases the probability of the supply of trade credit by about 15.4 percent. The ATEC indicates that firms with digital payment platforms increased their probability of engaging in trade credit demand by 25.9 percent and trade credit supply by 15.8 percent compared to firms that did not use digital payment platforms. The discussion follows that firms that had digital payment platforms were more likely to engage in trade credit activities than firms that did not use digital payment platforms. This dynamic is attributed to the fact that the use of digital payment platforms ensures efficiency and convenience in transactions, ensures credibility and security of transactions, offers better tools for cash flow management, and also forms part of a broader trend of technology adoption among firms.

**Table 4:** Treatment Effects of digital money on TC demand and supply.

Treatment Effects	Trade credit demand	Trade credit supply
Average Treatment Effect (ATE)	0.240** (0.096)	0.154*** (0.010)
Average Treatment Effect on the Treated (ATET)	0.259** (0.105)	0.158*** (0.011)

Robust Standard errors in parentheses.

*p < 0.05, **p < 0.01, ***p < 0.001.

#### 4.1.4 Average marginal effects- decomposition into direct and indirect effects

One way to split a predictor's marginal effect into direct and indirect effects is to use recursive bivariate probit estimation. The influence of a variable on a firm's choice to either supply or demand trade credit is measured by the direct effect. On the other hand, the indirect effect identifies the effects of a variable on a firm's decision to either demand for or supply trade credit through its effect on the use of digital payment platforms. The total effect of the variable on the dependent variable is the sum of the direct and indirect effects. Table 5 shows the decomposed marginal effects of the explanatory variables on the dependent variables.

**Table 5:** Marginal effects.

Effects	Trade credit demand			Trade credit supply		
	Direct	Indirect	Total	Direct	Indirect	Total
Age	−0.0007 (0.001)	0.001*** (0.0001)	0.001*** (0.0001)	0.008*** (0.002)	0.001 (0.001)	0.009*** (0.003)
Account	0.013 (0.022)	0.064*** (0.016)	0.077*** (0.012)	0.041* (0.022)	0.081*** (0.016)	0.122*** (0.016)
Business type						
Manufacturing	−0.018 (0.015)	0.027** (0.011)	0.010 (0.016)	0.021 (0.022)	0.033*** (0.012)	0.058** (0.023)
Services	−0.069*** (0.012)	0.024** (0.011)	−0.048*** (0.012)	−0.084*** (0.016)	0.028** (0.011)	−0.061*** (0.017)
Sales	0.001 (0.001)	0.001** (0.001)	0.001** (0.001)	0.001*** (0.0001)	0.001*** (0.001)	0.001*** (0.001)
Experience	0.001 (0.001)	−0.001 (0.001)	0.0003 (0.001)	−0.003 (0.002)	−0.001 (0.001)	−0.003 (0.002)
Education						
Basic	−0.055*** (0.020)	0.014* (0.008)	−0.027 (0.018)	−0.015 (0.019)	0.022* (0.012)	0.009 (0.021)
Secondary	−0.111*** (0.028)	0.075*** (0.020)	−0.038* (0.021)	−0.009 (0.028)	0.094*** (0.021)	0.087*** (0.026)
Tertiary	−0.077** (0.032)	0.062*** (0.021)	−0.005 (0.029)	−0.004 (0.034)	0.081*** (0.024)	0.079** (0.039)
Loan	0.135*** (0.016)	0.026** (0.012)	0.162*** (0.016)	0.129*** (0.021)	0.040** (0.016)	0.169*** (0.024)
Internet	0.042** (0.019)	0.050*** (0.015)	0.091*** (0.018)	0.057*** (0.022)	0.067*** (0.019)	0.124*** (0.025)
Operation hours	−0.0001 (0.0001)	0.0001* (0.0001)	0.0002 (0.0002)	0.001*** (0.0001)	0.0003** (0.0001)	0.001*** (0.0002)
Pay		0.038*** (0.008)	0.038*** (0.008)		0.055*** (0.011)	0.055*** (0.011)

Robust Standard errors in parentheses.

*p < 0.05, **p < 0.01, ***p < 0.001.

For instance, the age of a firm does not directly affect the demand for trade credit but does so through its effect on the use of digital payment platforms. Thus, increasing the age of the firm indirectly increases the firm's probability of involving itself in trade credit by about 0.001. However, increasing the age of the firm directly increases the probability of being involved in the supply of trade credit by about 0.008 and indirectly by about 0.001. Keeping accounting records indirectly increases the probability of trade credit demand by 0.064 and trade credit supply by 0.081, respectively. Manufacturing firms indirectly increase the probability of trade credit demand by about 0.027. The indirect effect outweighs the direct effect and, thus, results in a positive total effect.

Higher education directly reduces the probability of engaging in trade credit demand but directly increases the probability of trade credit demand. Likewise, higher education indirectly reduces the probability of a trade credit supply. Having a credit facility directly increases the probability of trade credit demand by about 0.135 and indirectly by about 0.026. Also, having a credit facility directly increases the probability of trade credit supply by about 0.129 and indirectly by about 0.040.

### 4.2 Robustness checks

In Table 6, we test for the robustness of previous results using a classical probit model where the possible correlations between the error terms are accounted for. The result of the probit confirms earlier results from the recursive bivariate probit. It indicates that the use of digital payment platforms increases the probability of firms’ trade credit demand and supply by 0.0666 and 0.182, respectively. Other variables that affect the demand and supply of trade credit are keeping accounting records, service firms, the education of the owner of the firm, credit facilities, the use of the internet and social media for marketing, and the hours of operations in a month.

**Table 6:** Results of binary probit.

Variables	Trade credit demand		Trade credit supply	
	Coefficient	dydx	Coefficient	dydx
dm	0.320***	0.0666***	0.601***	0.182***
	(0.0758)	(0.0157)	(0.0635)	(0.0183)
age	0.00445	0.000925	0.0333***	0.0101***
	(0.00801)	(0.00167)	(0.00743)	(0.00223)
account	0.312***	0.0648***	0.181**	0.0548**
	(0.0763)	(0.0158)	(0.0661)	(0.0199)
Business type				
Manufacturing	−0.0951	−0.0211	0.0867	0.0281
	(0.0916)	(0.0198)	(0.0792)	(0.0259)
Services	−0.512***	−0.0940***	−0.506***	−0.142***
	(0.0953)	(0.0152)	(0.0766)	(0.0196)
Sales	−3.47e-09	−7.22e-10	2.54e-08	7.70e-09
	(2.25e-08)	(4.69e-09)	(3.49e-08)	(1.06e-08)
Experience	0.0127	0.00263	−0.00933	−0.00282
	(0.00752)	(0.00156)	(0.00713)	(0.00216)
Education				
Basic	−0.264**	−0.0619*	−0.0801	−0.0246
	(0.0977)	(0.0242)	(0.0880)	(0.0272)
Secondary	−0.480***	−0.103***	−0.0946	−0.0289
	(0.115)	(0.0259)	(0.100)	(0.0307)
Tertiary	−0.267	−0.0624	−0.127	−0.0385
	(0.155)	(0.0351)	(0.137)	(0.0412)
Loan	1.042***	0.217***	0.657***	0.199***
	(0.102)	(0.0201)	(0.100)	(0.0296)
Internet	0.395***	0.0821***	0.337***	0.102***
	(0.105)	(0.0217)	(0.0962)	(0.0289)
Operation hours	−0.000237	−0.0000493	0.00322**	0.000975**
	(0.00115)	(0.000239)	(0.00103)	(0.000311)
C	−1.229***		−1.250***	
	(0.116)		(0.106)	
*N*	2458	2458	2460	2460
*R*^2^	0.1572		0.1341	

Robust Standard errors in parentheses.

*p < 0.05, **p < 0.01, ***p < 0.001.

## 5 Conclusion and recommendations

Understanding the relationship between the use of digital payment platforms and the decision to participate in the demand or supply of trade credit is crucial to finding the various sources of financing for informal firms and a broader objective of achieving financial inclusion for informal firms in Ghana. We argue that firms can mitigate the problem of access to finance using trade credit arrangements in a financially constraint environment. This study uses a recursive bivariate probit to determine the effect of the use of digital payment platforms on informal firms’ demand for and supply of trade credit in Ghana. Data from the World Bank enterprise survey data for informal firms is used for the purpose of the study. The study indicates that about 13.83 percent of informal firms receive trade credit from suppliers, and another 26.89 percent supply trade credit to customers. Also, about 49.6 percent of firms use digital payment platforms for their business transactions. The study also concludes that the use of digital payment platforms increases the probability of a firm being involved in the demand for and supply of trade credit. The use of digital payment platforms increases the efficiency, convenience, and security of transactions. It could also reduce the cost of transactions associated with financial transactions and enable transactions between firms and customers at distant locations. Other variables, including the age of the firm, the keeping of accounting records, volumes of sales, the experience of the owner, credit facilities, the use of the internet for social media marketing, and the number of operating hours, all significantly influence the decision to be involved in the demand and supply of trade credit. Our results are robust compared to other estimation techniques.

Given that trade credit is an innovative financing source for informal firms, especially when access to formal credit financing is increasingly becoming a concern, it is imperative that conscious efforts be made to increase the activities of trade credit. The results of the study have important implications for informal firms both in Ghana and beyond as digital payment platforms could contribute to the broader objectives of achieving financial inclusion. Firms that may have limited access to credit from formal financial institutions can leverage trade credit arrangements with suppliers and customers, thereby enhancing their financial resilience and business opportunities.

Trade credit is an important source of financing for firms especially those that lack access to credit from formal financial intermediaries. The results have demonstrated that one of the ways of promoting the use of trade credit in business transactions is through the availability and use of digital platforms. Therefore, it is recommended that firms and their suppliers and customers should adopt these digital payment platforms in order to facilitate their use of trade credit in business transactions. In order to encourage informal firms to use these digital payment platforms, government through policy should lower the transaction cost (e-levy and service charges) associated with the use of these digital platforms.

Policy from both firms and regulators should be focused on consciously pushing an agenda for the digitalization of informal firms. This could be facilitated by the government through investing in digital infrastructure to expand the reach and reliability of digital services. Also, policy should focus on reducing the costs and risk associated with technology adoption to facilitate the digitalization drive. This will improve financial transactions for businesses. Also, a regulatory environment that promotes business trust and the appropriate use of digital payment platforms should be ensured. This includes steps to guarantee data protection, security, and ethical business conduct within the ecosystem of digital payments.

Further studies could replicate this study for formal firms in Ghana based on the availability of current data. Also, further studies could evaluate the regulatory barriers and the policy environments that could hinder the digitalization (including, the rules governing the adoption of technology, the cost of technology) drive of firms and how that determines the adoption of digital payment platforms and, invariably, the use of trade credit.

## Data availability statement

Data will be made available upon request.

## CRediT authorship contribution statement

**Mohammed Gbanja Abdulai:** Writing – original draft, Methodology, Formal analysis. **Stanley Kojo Dary:** Writing – review & editing, Methodology. **Paul Bata Domanban:** Writing – review & editing, Methodology, Conceptualization.

## Declaration of competing interest

The authors declare that they have no known competing financial interests or personal relationships that could have appeared to influence the work reported in this paper.
